# Effect of Steroid Hormones, Prostaglandins (E2 and F2α), Oxytocin, and Tumor Necrosis Factor Alpha on Membrane Progesterone (P4) Receptors Gene Expression in Bovine Myometrial Cells

**DOI:** 10.3390/ani12040519

**Published:** 2022-02-19

**Authors:** Magdalena K. Kowalik, Karolina Dobrzyn, Jaroslaw Mlynarczuk, Robert Rekawiecki

**Affiliations:** Institute of Animal Reproduction and Food Research of the Polish Academy of Sciences, Tuwima 10, 10-747 Olsztyn, Poland; k.dobrzyn@pan.olsztyn.pl (K.D.); j.mlynarczuk@pan.olsztyn.pl (J.M.); r.rekawiecki@pan.olsztyn.pl (R.R.)

**Keywords:** membrane progesterone receptors, non-genomic action, PGRMC, mPR, myometrium, uterus, cow

## Abstract

**Simple Summary:**

The myometrium is one of the layers of the uterus. It consists of smooth muscle and can therefore stretch during pregnancy and contract during parturition. Thus, it can assist oocyte fertilization and its transport in the fallopian tube as well as the blastocyst implantation process. Myometrial function is regulated by the activation of several signal transduction pathways and the expression of many genes. Among them are non-genomic membrane progesterone (P4) receptors such as progesterone receptor membrane components (PGRMC) 1 and 2 and membrane progestin receptors (mPR) alpha (mPRα), beta (mPRβ), and gamma (mPRγ). Their improper action may cause disturbances in fertilization and the correct course of the estrous cycle and pregnancy. The results of this study indicate the possible role of P4, estradiol (E2), prostaglandins (PG) E2 and F2α, oxytocin (OT), and tumor necrosis factor alpha (TNFα) in the regulation of membrane P4 receptor gene expression. This suggests that the local hormonal milieu may influence the activity of these receptors and P4 action in myometrial cells during the estrous cycle. Therefore, elucidation of the mechanisms by which the action of membrane P4 receptors in the myometrium is regulated may be an important element in understanding the proper functioning of the uterus and can help to reduce abnormalities in the course of pregnancy in cows.

**Abstract:**

Myometrium tissue shows the expression of non-genomic membrane progesterone (P4) receptors, such as progesterone receptor membrane components (PGRMC) 1 and 2 and membrane progestin receptors (mPR) alpha (mPRα), beta (mPRβ), and gamma (mPRγ). Their variable expression in the bovine uterus during the estrous cycle and early pregnancy suggests that ovarian steroids and luteotropic and/or luteolytic factors may regulate the expression of these receptors in the myometrium. Therefore, this study aimed to examine the effect of P4, estradiol (E2), P4 with E2, prostaglandins (PG) E2 and F2α, oxytocin (OT), and tumor necrosis factor α (TNFα) on the gene expression of PGRMC1, PGRMC2, serpine-1 mRNA-binding protein (SERBP1), and mPRα, mPRβ, and mPRγ in bovine myometrial cells from days 6 to 10 and 11 to 16 of the estrous cycle. The PGE2 concentration and mRNA expression were determined by EIA and real-time PCR, respectively. The data indicated that P4 and E2 can affect the mRNA expression of all studied receptors and SERPB1. However, PGE2, OT, and TNFα could only modulate the expression of PGRMC1, PGRMC2, and SERPB1, respectively. Steroids/factors changed the expression of PGRMC and mPR genes depending on the dose, the stage of the estrous cycle, and the types of receptors. This suggests that the local hormonal milieu may influence the activity of these receptors and P4 action in myometrial cells during the estrous cycle.

## 1. Introduction

Progesterone (P4) is one of the key regulators in the mammalian reproductive tract. The main sources of P4 are the corpus luteum (CL), ovarian follicles, and placenta [1,2]. This steroid hormone regulates the estrous cycle, creates suitable conditions for embryo implantation in the uterus, and ensures the maintenance of pregnancy [1]. Most effects of P4 are mediated through its nuclear progesterone receptor (PGR) and have a genomic effect [3]. However, some cellular effects of P4 have a non-genomic effect and are generated through its membrane receptors [4,5,6]. Currently, two groups of membrane P4 receptors are known. The first of these is the progesterone receptor membrane components (PGRMC), including types 1 and 2, which belong to the membrane-associated progesterone protein (MAPR) family [7]. The second group consists of membrane progestin receptors (mPR) and contains three subtypes, i.e., alpha (mPRα), beta (mPRβ), and gamma (mPRγ) [5,8], but each isoform is encoded by a different gene [9]. The mPRs belong to the progestin and adipoQ receptor (PAQR) protein family [7,10].

The expression of PGRMC1 and PGRMC2 (mRNA and/or protein) was detected in the bovine endometrium [11], myometrium [12,13], oviduct [14,15], and CL [12,16]. It has been shown that PGRMC1 regulates the intracellular metabolism of cholesterol, influences the rate of steroidogenesis [17,18], inhibits the motoric activity of the myometrium [19], and supports oocyte maturation [20]. However, to be fully active, this receptor binds to its co-receptor, serpine-1 mRNA-binding protein (SERBP1), and forms a P4 receptor–membrane complex [5]. Far fewer data are available on the PGRMC2 receptor. It is known that it takes part in regulating the secretory and motoric activity of the oviduct [14,15]. Moreover, changes in its quantity and activity were found in cases of premature births [21].

The presence of mPRs in the bovine reproductive tract was demonstrated in the CL [22], oviduct [15], and uterus [23]. This points to their possible involvement in promoting apoptosis in luteal cells and CL regression [5,24] as well as participation in oocyte fertilization and its transport in the fallopian tube [25,26,27]. In the uterus, mPRs support the blastocyst implantation process, pregnancy maintenance, and birth [28,29,30].

The myometrium is composed of myometrial smooth muscle cells and is the tissue responsible for uterine contractions. It was found that in the myometrium, P4 may act through the non-genomic mechanism that directly affects myometrial contraction by modulating intracellular signal transduction pathways [29,31] or inhibiting calcium influx [28]. These processes may involve activation of membrane P4 receptors, which are expressed in myometrial cells in women [19,29,31,32], cows [11,13,23] and mice [33]. This suggests that membrane P4 receptors are relevant for myometrium contractility, which is important for proper embryo development and parturition.

During the estrous cycle, the myometrium is influenced by many hormones, produced locally (mainly in the endometrium) and synthesized in the CL, that cause various changes in its motoric and secretory processes. These hormones also modulate the expression (mRNA/protein) of membrane P4 receptors [11,13,23], with the greatest changes observed between the 6th and 16th days of the cycle [11,13,23]. However, the factors regulating the course of the estrous cycle and influencing the expression of membrane P4 receptors in the bovine myometrium have not been identified so far. Therefore, the present study aimed to examine the influence of P4 and estradiol (E2) hormones both individually and combined, oxytocin (OT), prostaglandins (PG) E2 and F2α, and tumor necrosis factor alpha (TNFα) on the mRNA expression of PGRMC1, PGRMC2, SERBP1, mPRα, mPRβ, and mPRγ. Bovine myometrial cells from days 6 to 10 and 11 to 16 of the estrous cycle were used as the research model.

## 2. Materials and Methods

### 2.1. Tissue Collection

Uteri horns (n = 5), ipsilateral to the ovary with active CL, were collected from a local slaughterhouse and transported to the laboratory within 1 h in 0.9% NaCl (supplemented with 10 IU/mL penicillin, 100 μg/mL streptomycin, 2 μg/mL amphotericin, and 100 μg/mL L-glutamine), cooled to a temperature of 4–6 °C in an ice water bath. Uteri from 5 animals were collected for each phase of the estrous cycle. The days of the estrous cycle were assessed based on morphological observations of the ovaries (color, blood supply, size of the CL, and the diameter and size of ovarian follicles) and the uterus (color and fluffiness of the endometrium; the amount, color, consistency, and clarity of the mucus inside the uterine cavity; and the size and color of the uterine glands) as previously described [34,35]. Unless otherwise stated, all materials used in this study were purchased from Sigma (Poznan, Poland).

### 2.2. Isolation and Culture of Myometrial Cells

Myometrial cells from the horn ipsilateral to the ovary with CL were isolated using collagenase IV as described by Wrobel et al. [36] and Słonina et al. [13]. The cell concentrations and viability were determined using 0.04% trypan blue dye. Only cells with viabilities above 85% were used in experiments. The obtained cells were suspended at a concentration of 2.5 × 10^5^ cells/mL in DMEM/Ham’s F12 medium supplemented with 10% NCS, 40 ng/mL gentamicin (KRKA, Novo Mesto, Slovenia), and 20 μg/mL amphotericin. Then, the cell suspension was added to 6-well plates (Nunclon, NUNC, Roskilde, Denmark) at 2 mL/well. The cells were incubated for 96 h (Memmert INCO 180, Schwabach, Germany) to achieve approximately 80% confluence. The culture medium was changed every two days. Next, cells were washed two times, and the medium was replaced with M-199 without phenol red and supplemented with 0.1% BSA. Next, cells were stimulated for 6 h with P4 (10^−7^ M, 10^−6^ M, 10^−5^ M), E2 (10^−10^ M, 10^−9^ M, 10^−8^ M), P4/E2 (10^−6^/10^−9^ M, 10^−5^/10^−8^ M), OT (10^−7^ M), PGE2 (10^−6^ M), PGF2α (10^−6^ M), and TNFα (10^−4^ M). Doses of factors were based on preliminary studies and previous reports in the literature [37,38,39]. Cells were maintained in a humidified atmosphere of 95% O_2_ and 5% CO_2_ throughout the incubation time. Then, cells were harvested, mixed with TRIzol (A&A Biotechnology, Poland), placed into Eppendorf tubes, and stored at −20 °C for further analysis by PCR. One well in each plate was stimulated with arachidonic acid (AA; 10^−5^ M) as a positive control for PGE2 production. From this well, the medium was aspirated and preserved at −20 °C for the determination of PGE2 concentrations. All treatments were performed in triplicate in five separate experiments.

### 2.3. PGE2 Determination

Concentrations of PGE2 were determined by enzyme immunoassay (EIA) using a microplate reader (Multiscan EX, Labsystem, Helsinki, Finland). Horseradish peroxidase-labeled PGE2 and anti-PGE2 were used in final dilutions of 1:30,000 and 1:35,000, respectively. The standard curve ranged from 0.001 to 20.4 ng/mL. The intra- and inter-assay coefficients of variation were 1.79% and 5.44%, respectively. For further analysis, we used only cells obtained from the plate where the concentration of PGE2 in the positive control (which was AA) was higher compared with the untreated cells. The increase in PGE2 secretion from the cells incubated with AA indicated the viability of the cells and their potential responses to the factors evaluated (Figure S1, supplementary data).

### 2.4. Total RNA Isolation and cDNA Synthesis

Total RNA was extracted from myometrial cells using the Total RNA Prep Plus Kit (A&A Biotechnology, Gdańsk, Poland) according to the manufacturer’s instructions. Before use, RNA concentration and quality were determined using a Nanodrop 1000 spectrophotometer (Thermo Scientific, Waltham, MA, USA). Moreover, the RNA integrity number (RIN) was evaluated on the Bioanalyzer 2100 instrument (Agilent Technologies, Santa Clara, CA, USA). Samples were characterized by an RIN between 8 and 10. An amount of 1000 ng of each sample of total RNA was reverse transcribed (RT) into cDNA using M-MLV Reverse Transcriptase. First, 8 μL RNA was mixed with 1 μL DNAse and 1 μL of reaction buffer, and the sample was incubated for 10 min at room temperature. Then, 1μL EDTA and 1 μL oligodT RNA were added. The mixture was denatured at 70 °C for 10 min. Next, 2 μL 10× RT, 1 μL dNTP, 1 μL M-MLV, and 4 μL H_2_O were added to the reaction mixture. The total volume of the RT reaction mixture was 20 μL. The sample was placed in a thermal cycler at 37 °C for 50 min. The contained cDNA was then diluted in nuclease-free water (Promega, Madison, WI, USA) and stored at −20 °C for the real-time PCR procedure.

### 2.5. Real-Time PCR

Real-time PCR analysis was performed using the ABI 7900 real-time PCR System (Applied Biosystems, Foster City, CA, USA) for sequence detection with the Power SYBR Green PCR Master Mix (Applied Biosystems). Each PCR reaction sample contained 3 μL of diluted cDNA, 5 μL SYBR Green PCR Master Mix, and 1 μL of each of the primers at 200 pM. The primers used to amplify PGRMC1, PGRMC2, SERBP1, mPRα, mPRβ, mPRγ, and TBP (reference gene) were the same as those described by [11,23]. The housekeeping gene was chosen based on Rekawiecki et al. [40]. The primer sequences and sizes of the amplified fragments of all the transcripts are shown in Table 1. The real-time PCR protocol started with initial denaturation (10 min at 95 °C), followed by 40 cycles of denaturation, annealing, and elongation (1 min at 60 °C for each, except PGRMC2, for which a temperature of 58 °C was used). After each PCR reaction, melting curve analysis was performed to confirm single product amplification. All PCR reactions were performed in duplicate. The specificity of the product was also confirmed by electrophoresis using a 2% agarose gel (EuroGenTec, Koln, Germany). Data from the RT-PCR were normalized to the TBP mRNA content and expressed as arbitrary units. For the relative quantification of mRNA levels, Miner software was used [41].

### 2.6. Statistical Analysis

The real-time PCR results were analyzed using the real-time PCR Miner algorithm [41]. Statistical analyses were conducted using GraphPad Prism v. 8.0 software (GraphPad Software, San Diego, CA, USA). All experimental data are shown as the mean ± standard error of the mean (SEM), and differences were considered statistically significant at the 95% confidence level (*p* < 0.05). The analyses were performed using one-way ANOVA, followed by Bonferroni’s multiple comparison test after testing for normality.

## 3. Results

### 3.1. The Effect of Steroid Hormones on the mRNA Expression of PGRMC1 and PGRMC2

**Figure 1:**
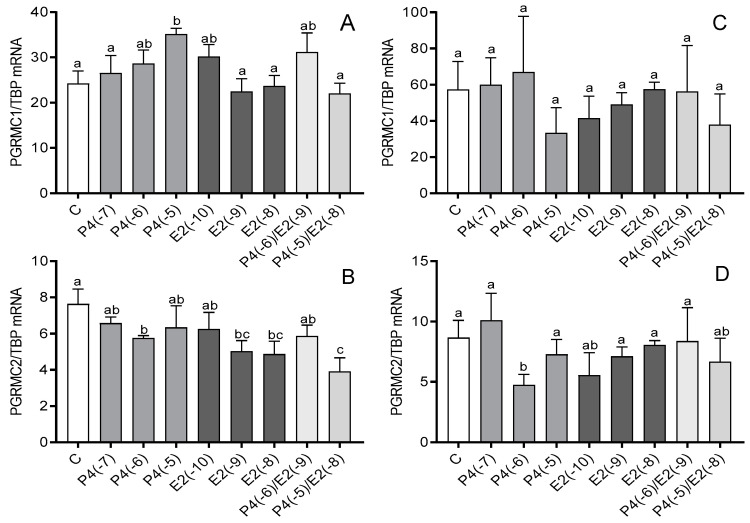
The influence of progesterone (P4; 10^−7^ M, 10^−6^ M, 10^−5^ M), estradiol (E2; 10^−10^ M, 10^−9^ M, 10^−8^ M), and P4 together with E2 (10^−6^/10^−9^ M, 10^−5^/10^−8^ M) on the mRNA expression of progesterone membrane component 1 (PGRMC1) (**A**,**C**) and PGRMC2 (**B**,**D**) in bovine myometrial cells from days 6 to 10 (**A**,**B**) and 11 to 16 (**C**,**D**) of the estrous cycle. Gene expression was determined by quantitative real-time PCR. Results are reported as mean ± SEM (n = 5). Bars with different letters (a, b, c) differ significantly (*p* < 0.05).

In cultures of myometrial cells from days 6 to 10 of the estrous cycle, increased PGRMC1 mRNA expression (*p* < 0.05) was noted after treatment with 10^−5^ M P4 (Figure 1A), while a 10^−6^ M dose of P4 decreased PGRMC2 mRNA expression (*p* < 0.05; Figure 1B). Additionally, 10^−9^ M and 10^−8^ M E2 decreased the mRNA expression of this gene compared to the control (*p* < 0.05). The same effect was observed after treatment with a combination of P4 (10^−5^ M) and E2 (10^−8^ M; *p* < 0.05; Figure 1B). In cultures of myometrial cells from days 11 to 16 of the estrous cycle, only P4 at a dose of 10^−6^ M caused a reduction in PGRMC2 mRNA expression (*p* < 0.05; Figure 1D).

### 3.2. The Effect of Prostaglandins (E2 and F2α), OT, and TNFα on the mRNA Expression of PGRMC1 and PGRMC2

**Figure 2:**
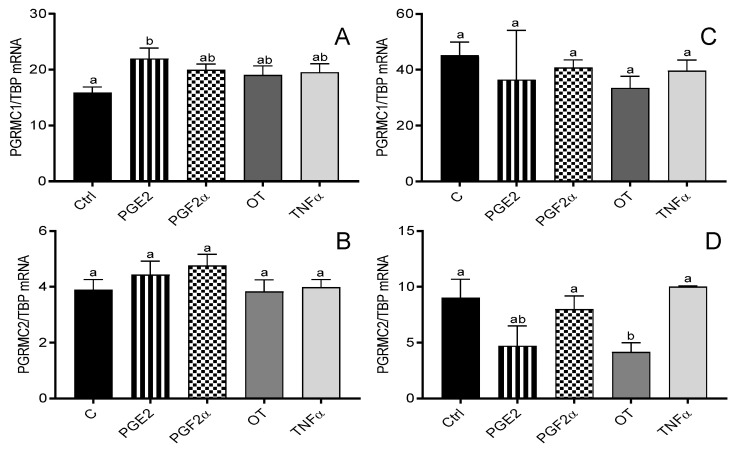
The influence of prostaglandin E2 (PGE2; 10^−6^ M), prostaglandin F2α (PGF2α; 10^−6^ M), oxytocin (OT; 10^−7^ M), and tumor necrosis factor alpha (TNFα; 10^−4^ M) on the mRNA expression of progesterone membrane component 1 (PGRMC1) (**A**,**C**) and PGRMC2 (**B**,**D**) in bovine myometrial cells from days 6 to 10 (**A**,**B**) and 11 to 16 (**C**,**D**) of the estrous cycle. Gene expression was determined by quantitative real-time PCR. Results are reported as mean ± SEM (n = 5). Bars with different letters (a, b) differ significantly (*p* < 0.05).

Only PGE2 (10^−6^ M) increased PGRMC1 mRNA expression in myometrial cells from days 6 to 10 of the estrous cycle (*p* < 005; Figure 2A), while OT (10^−7^ M) decreased PGRMC2 mRNA expression in cells from days 11 to 16 of the estrous cycle (*p* < 0.05; Figure 2D).

### 3.3. The Effect of Steroid Hormones, Prostaglandins (E2 and F2α), OT, and TNFα on the mRNA Expression of SERBP1

**Figure 3:**
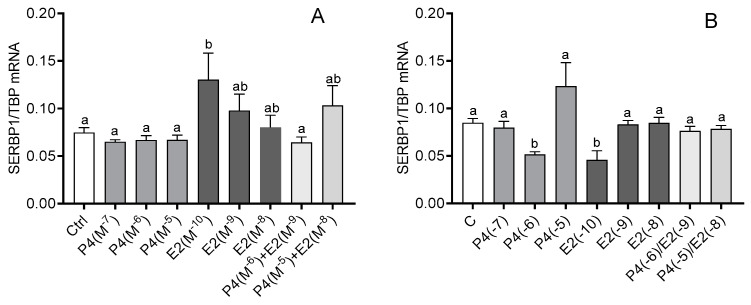
The influence of progesterone (P4; 10^−7^ M, 10^−6^ M, 10^−5^ M), estradiol (E2; 10^−10^ M, 10^−9^ M, 10^−8^ M), and P4 together with E2 (10^−6^/10^−9^ M, 10^−5^/10^−8^ M) on the mRNA expression of serpine-1 mRNA-binding protein (SERBP1) in bovine myometrial cells from days 6 to 10 (**A**) and 11 to 16 (**B**) of the estrous cycle. Gene expression was determined by quantitative real-time PCR. Results are reported as mean ± SEM (n = 5). Bars with different letters (a, b) differ significantly (*p* < 0.05).

**Figure 4:**
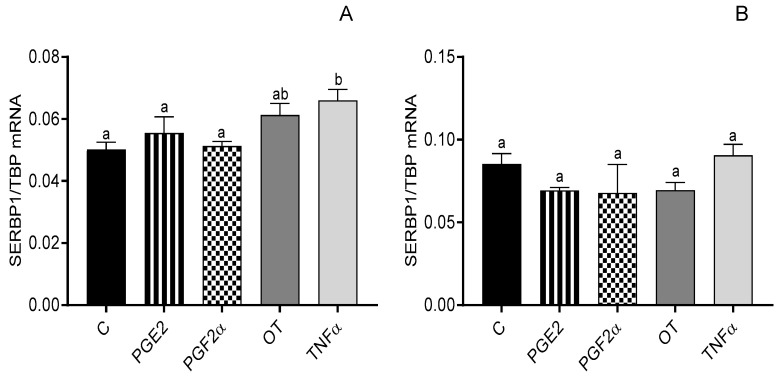
The influence of prostaglandin E2 (PGE2; 10^−6^ M), prostaglandin F2α (PGF2α; 10^−6^ M), oxytocin (OT; 10^−7^ M), and tumor necrosis factor alpha (TNFα; 10^−4^ M) on the mRNA expression of serpine-1 mRNA-binding protein (SERBP1) in bovine myometrial cells from days 6 to 10 (**A**) and 11 to 16 (**B**) of the estrous cycle. Gene expression was determined by quantitative real-time PCR. Results are reported as mean ± SEM (n = 5). Bars with different letters (a, b) differ significantly (*p* < 0.05).

In myometrial cells from days 6 to 10 of the estrous cycle, the mRNA expression of SERBP1 increased after E2 treatment at a dose of 10^−10^ M (*p* < 0.05; Figure 3A), but in myometrial cells from days 11 to 16 of the estrous cycle, P4 at a dose of 10^−6^ M and E2 at a dose of 10^−10^ M caused a decrease in SERBP1 mRNA expression (*p* < 0.05; Figure 3B). SERBP1 mRNA expression was increased only after TNFα treatment (*p* < 0.05; Figure 4A).

### 3.4. The Effect of Steroid Hormones, Prostaglandins (E2 and F2α), OT, and TNFα on the mRNA Expression of mPRα, mPRβ, and mPRγ

**Figure 5:**
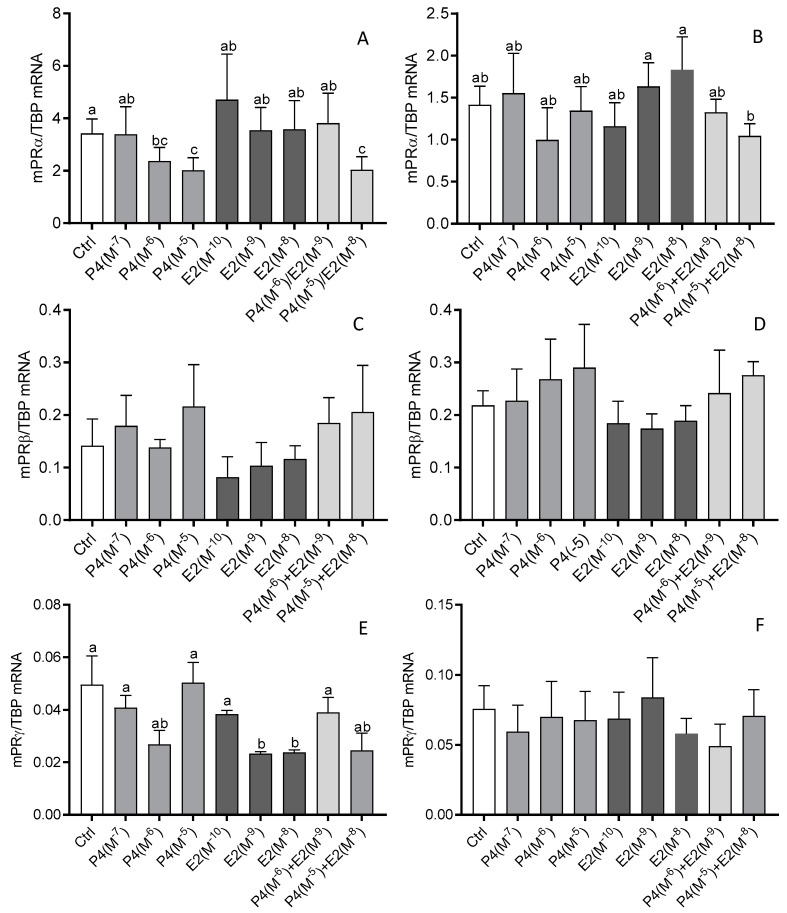
The influence of progesterone (P4; 10^−7^ M, 10^−6^ M, 10^−5^ M), estradiol (E2; 10^−10^ M, 10^−9^ M, 10^−8^ M), and P4 together with E2 (10^−6^/10^−9^ M, 10^−5^/10^−8^ M) on the mRNA expression of membrane progestin receptor α (mPRα) (**A**,**B**), mPRβ (**C**,**D**), and mPRγ (**E**,**F**) in bovine myometrial cells from days 6 to 10 (**A**,**C**,**E**) and 11 to 16 (**B**,**D**,**F**) of the estrous cycle. Gene expression was determined by quantitative real-time PCR. Results are reported as mean ± SEM (n = 5). Bars with different letters (a, b, c) differ significantly (*p* < 0.05).

In myometrial cells from days 6 to 10 of the estrous cycle, 10^−6^ M and 10^−7^ M of P4 caused a decrease in mPRα mRNA expression (*p* < 0.05; Figure 5A). The same effect was observed after treatment of these cells with a mixture of P4 (10^−5^ M) and E2 (10^−8^ M; *p* < 0.05; Figure 5A). On the other hand, the expression of mPRγ mRNA was reduced after E2 treatment at doses of 10^−9^ M and 10^−8^ M (*p* < 0.05; Figure 5C). None of the used hormones or their mixtures affected the expression of mPRβ mRNA in myometrial cells obtained on days 6–10 and 11–16 of the estrous cycle (*p* > 0.05; Figure 5C,D). In myometrial cells from days 11 to 16 of the estrous cycle (*p* < 0.05; Figure 5D), we noted only a difference in mPRα mRNA expression between E2 at a dose of 10^−9^ M and 10^−8^ M and a mixture containing P4 (10^−5^ M) and E2 (10^−8^ M; *p* < 0.05; Figure 5B).

None of the other factors (PGE2, PGF2α, OT, and TNFα) altered the mRNA expression of any of the mPR subtypes in myometrial cells obtained on days 6–10 and days 11–16 of the estrous cycle (*p* > 0.05; Figure S2, supplementary data).

## 4. Discussion

In the present study, we demonstrated the mRNA expression of all studied membrane P4 receptors and SERPB1 protein in bovine myometrial cells from days 6 to 16 of the estrous cycle and the hormonal regulation of PGRMC and mPR genes in these cells. We found that P4 and E2 can affect the mRNA expression of all membrane P4 receptors and SERPB1; however, the other regulatory factors used in this study, including PGE2, OT, and TNFα, may only regulate the expression of PGRMC1, PGRMC2, and SERPB1, respectively. The effect of the tested steroid hormones and factors depends on their concentrations, the incubation time, the phase of the estrous cycle, and the type of receptor.

Expression of mRNA for membrane P4 receptors was found in human, mouse, and sheep uteri [28,29,31,33,42], but despite their potential importance, limited data have presented the factors which may regulate the expression of mPRs in the uterus of cows. Moreover, the role of steroid hormones in the regulation of mRNA expression for membrane P4 receptors in the reproductive tract is not clear. Previous studies [33] have shown that P4 stimulates PGRMC1 and PGRMC2 in the mouse uterus. Other studies [43,44] pointed out that the expression of PGRMC1 can be inhibited by P4 as well as by E2. Our results have shown that the effect of P4 depends on the phase of the estrous cycle and the dose of the used steroids and can be different for PGRMC1 and PGRMC2. During the time of rapid increase in P4 release from the CL (days 6–10 of the estrous cycle), the expression of PGRMC1 mRNA increased, while the expression of PGRMC2 mRNA decreased even if P4 was added jointly with E2 to cultured cells. Similarly, the effect of P4 on PGRMC2 was also observed in myometrial cells obtained on days 11–16 of the estrous cycle. On the other hand, E2 inhibited the mRNA expression of PGRMC2 in the earlier phase of the estrous cycle. The observed changes suggest that the expression of both PGRMCs is regulated mainly by P4; however, in the case of PGRMC2, it can be supposed that the combined effect of both P4 and E2 may regulate its mRNA expression. It should be pointed out that the non-genomic action of P4 can occur partly through E2, which regulates the activity of nuclear P4 receptors [45] and subsequently activates the intracellular protein kinase pathways in the cell [8]; this way, both steroids may jointly modulate the gene expression of membrane P4 receptors through the regulation of PGR. However, this combined effect of both steroids may be dependent on the E2:P4 ratio and the number and proportion of E2 and P4 receptors in the cell/tissue. This suggestion may explain the differences in the regulation of membrane P4 receptor expression in the different types of cells or tissues.

Our studies have shown that non-steroidal factors such as PGE2 and OT can regulate PGRMC1 and PGRMC2 mRNA expression, respectively. It is clear that these effects are part of a broader mechanism of regulation of the physiological status of the myometrial tissue during the estrous cycle. In the first part of the estrous cycle (P4 part), PGE2 as a luteotropic factor increases the sensitivity of the myometrium to P4 influence by increasing the expression of PGRMC1 mRNA. If fertilization and the development of pregnancy do not occur, before the next estrous cycle, the endo- and myometrium are rebuilt. In this process, the main regulatory factor is E2 and high sensitivity of myometrial cells to P4 is not necessary. Hence, OT may decrease the expression of PGRMC2 mRNA, and this may reduce the impact of P4 on these cells during this period.

The obtained results showed that E2 had opposing effects on the mRNA expression of SERPB1 in both studied parts of the estrous cycle. This is possible because E2 often shows a biphasic effect on some physiological characteristics in the regulation of reproductive processes. A very similar influence of E2 was described by Voss and Fortune [46] on oxytocin secretion by bovine granulosa cells. However, the exact mechanism of these biphasic E2 effects has not been explained. On the other hand, P4 decreased the mRNA expression of SERBP1 in bovine myometrial cells only on days 11–16, suggesting that the used dose of P4 (which is lower than the average P4 concentration in bovine serum during diestrus) regulates the expression of this factor. However, simultaneously treating cells with a mixture of P4 and E2 erased all the effects that were observed after treatment with E2 or P4 alone. Nevertheless, this does not exclude the thesis put forward earlier that the mRNA expression of SERBP1 can be regulated by the combined effect of P4 and E2 in specific proportions.

It should be pointed out that the most changes in PGRMC2 and SERPB1 mRNA expression under the influence of steroid hormones and other factors used in the experiments occurred on days 11–16 of the cycle and were characterized by inhibition of expression, which illustrates the extinction of the progesterone phase during the estrous cycle. Moreover, they indicate a different way of regulating the activity of receptors of the PGRMC type. The expression of the mRNA of the PGRMC2 receptor is directly inhibited by steroid hormones as well as PGE2 and OT. On the other hand, in the case of the PGRMC1 receptor, the mRNA expression of its cofactor, SERBP1, is inhibited, which is necessary to maintain its full activity [5]. Thus, changes in cofactor expression may influence the activity of this receptor in myometrial cells.

Moreover, in this study, we showed that TNFα increases the expression of SERBP1 mRNA in bovine myometrial cells on days 6–10. The action of TNFα in the reproductive system is multidirectional [47]. It has been shown that this factor is involved in the regulation of immunologic, inflammatory, or reparative responses and may control PG synthesis in the endometrium in cattle [48,49], thereby playing a role in regulating uterine function throughout the estrous cycle [47]. In the early follicular phase, it may cause atresia of the ovarian follicle, while in the early luteal phase, it exhibits luteotropic and then luteolytic effects [50]. TNFα has been shown to stimulate PGF2α secretion from the endometrium during the follicular phase as well as in the late luteal phase, suggesting that this cytokine is a factor initiating luteolysis in cattle [51]. The increase in expression of SERBP1 under the influence of TNFα may be part of the mechanism to prevent a fast decline in the sensitivity of myometrial cells to P4. It could prevent a rapid increase in uterine motility and thus facilitate the implantation of the blastocyst. On the other hand, these data may suggest that TNFα, by affecting the expression of the SERBP1 gene, can regulate the expression of the PGRMC1 gene. Thus, TNFα may be involved in the expression of the SERBP1 protein to influence the activity of the PGRMC1–SERBP1 protein complex in cattle.

The results obtained in this study revealed that only steroid hormones change the mRNA expression of mPRα and mPRγ. Moreover, none of the examined factors changed the expression of the mPRβ receptor. Regarding the receptors of this group, changes in mRNA expression were recorded on days 6–10 of the estrous cycle, contrary to the PGRMC-type receptors. Furthermore, different steroid hormones caused inhibition of mPRα and mPRγ expression, namely P4 and E2, respectively. This indicates that both types of membrane P4 receptors may have a different mechanism regulating their expression in bovine myometrial cells and may have a distinct role in the myometrium. This suggestion was partly confirmed by data obtained from human myometrium samples [31], where mPRα and mPRβ expression was shown to be induced by P4 and E2, respectively. Nevertheless, the effect of steroid hormones on the expression of the membrane P4 receptors seems to depend on the type of receptor, target tissues, and animal model [31,52,53]. In addition, other factors such as prolactin [54] or interleukin 1β [55] may control the level of membrane P4 receptor mRNA; thus, further detailed studies are required.

The collected data indicate that the regulation of the expression of membrane P4 receptors in the myometrium of cows can be influenced by various factors and changes during the studied period of the estrous cycle. The steroid hormones (P4 and E2), OT, PGE2, and TNFα could be the factors regulating mRNA expression in a stage- and dose-dependent manner. Although our observations suggest the possible influence of steroids, OT, and PGE2 on the mRNA expression of membrane P4 receptors and of TNFα on SERBP1 mRNA expression in bovine myometrial cells, further studies to identify the exact relationship between the membrane P4 receptor content and steroids/other factors in bovine myometrial cells are needed.

## 5. Conclusions

In conclusion, this study contributes new findings on the in vitro regulation of membrane P4 receptor expression by steroid hormones, PGE2, OT, and TNFα in the bovine myometrium during the estrous cycle. The regulatory mechanisms underlying PGRMC and mPR genes’ expression are complex and could be regulated by various physiological factors, whose action/effect depends on the time and dose of the agent and the hormonal status of the animal. The presence of membrane P4 receptors in bovine myometrial cells suggests that these receptors are involved in the regulation of the secretory and contractility functions of the myometrium during the estrous cycle. The unaffected regulation of uterine motility is one of the crucial factors for the course of the estrous cycle as well as fertilization, implantation, and maintenance of pregnancy; therefore, these results may be used as a basic reference for further studies in this area.

## Figures and Tables

**Table 1:** Forward (F) and reverse (R) primer sequences, amplicon lengths, and GenBank accession numbers of genes used during real-time PCR analysis. Amplicons’ sizes are expressed in the number of base pairs (bp). PGRMC1—progesterone membrane component 1; PGRMC2—progesterone membrane component 2; SERBP1—serpine-1 mRNA-binding protein; mPRα—membrane progestin receptor α; mPRβ—membrane progestin receptor β; mPRγ—membrane progestin receptor γ; and TBP—TATA-binding protein.

Gene	Primers Forward (F)/Reverse (R)	Amplicon Length (bp)	GenBank Accession No.
PGRMC1	F: TCTTCAGGGGTGTGTGTGAA R: CATTGTCCTGTGCTCTTTGG	266	NM_001075133
PGRMC2	F: TGCCTCTTTGCCTCGTATGA R: GAGGCATCCCTACCAGCAAAT	179	NM_001099060
SERBP1	F: AGCTCAGACCAACTCCAATGC R: CGGCTCAGACCTTCTTTCTTCA	149	NM_001046449
mPRα	F: CCGGCGGTCCATCTATGA R: CCACCCCCTTCACTGAGTCTT	159	NM_001038553
mPRβ	F: TGCCCCTGCTCGTCTATGTC R: CCCACGTAGTCCACGAAGTAGAA	120	NM_001101135
mPRγ	F: GGTTCTTCTCGTGGAGGTTTGT R: GTTCCTGGACATGGAGCTGAA	151	XM_005193580
TBP	F: CAGAGAGCTCCGGGATCGT R: CACCATCTTCCCAGAACTGAATAT	194	NM_001075742.1

## Data Availability

All the data presented in this study are included in the article and supplementary files.

## Supplementary Materials

The following supporting information can be downloaded at: https://www.mdpi.com/article/10.3390/ani12040519/s1. The file contains two complementary Figures S1 and S2.
