# Supplementary material for: Effect of Steroid Hormones, Prostaglandins (E2 and F2α), Oxytocin, and Tumor Necrosis Factor Alpha on Membrane Progesterone (P4) Receptors Gene Expression in Bovine Myometrial Cells
Source: Animals (Basel). 2022 Feb 19;12(4):519. doi: 10.3390/ani12040519 (PMC8868417; doi:10.3390/ani12040519)
Supplement: Supplementary file 1 [file animals-12-00519-s001.zip › animals-1513573-supplementary.pdf]

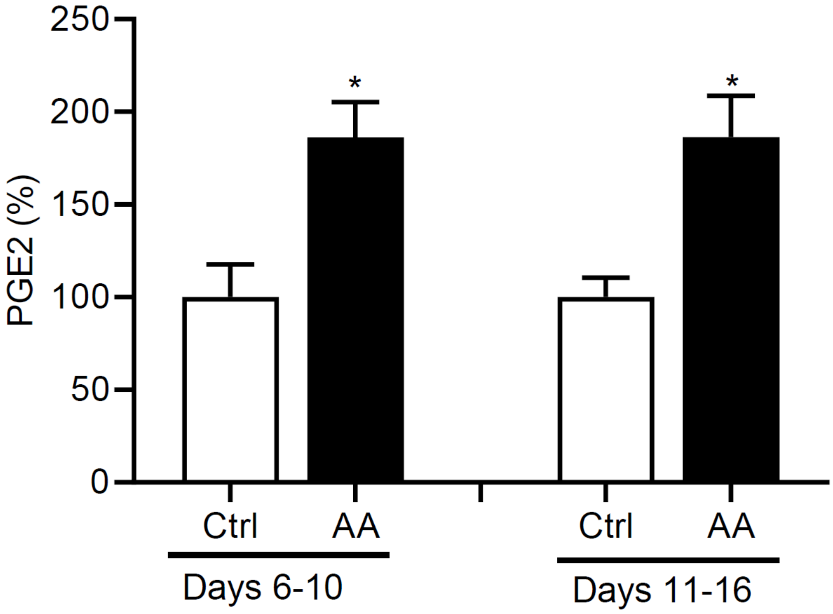

Figure S1. Mean (±S.E.M.) PGE2 concentrations in medium after supplementation of myometrial cells on Days 6 to 10 and 11 to 16 of the estrous cycle with arachidonic acid (AA; 10−5 M) compared to untreated cells (Ctrl; n = 5). Values with different superscripts are significant at *P*<0.01.

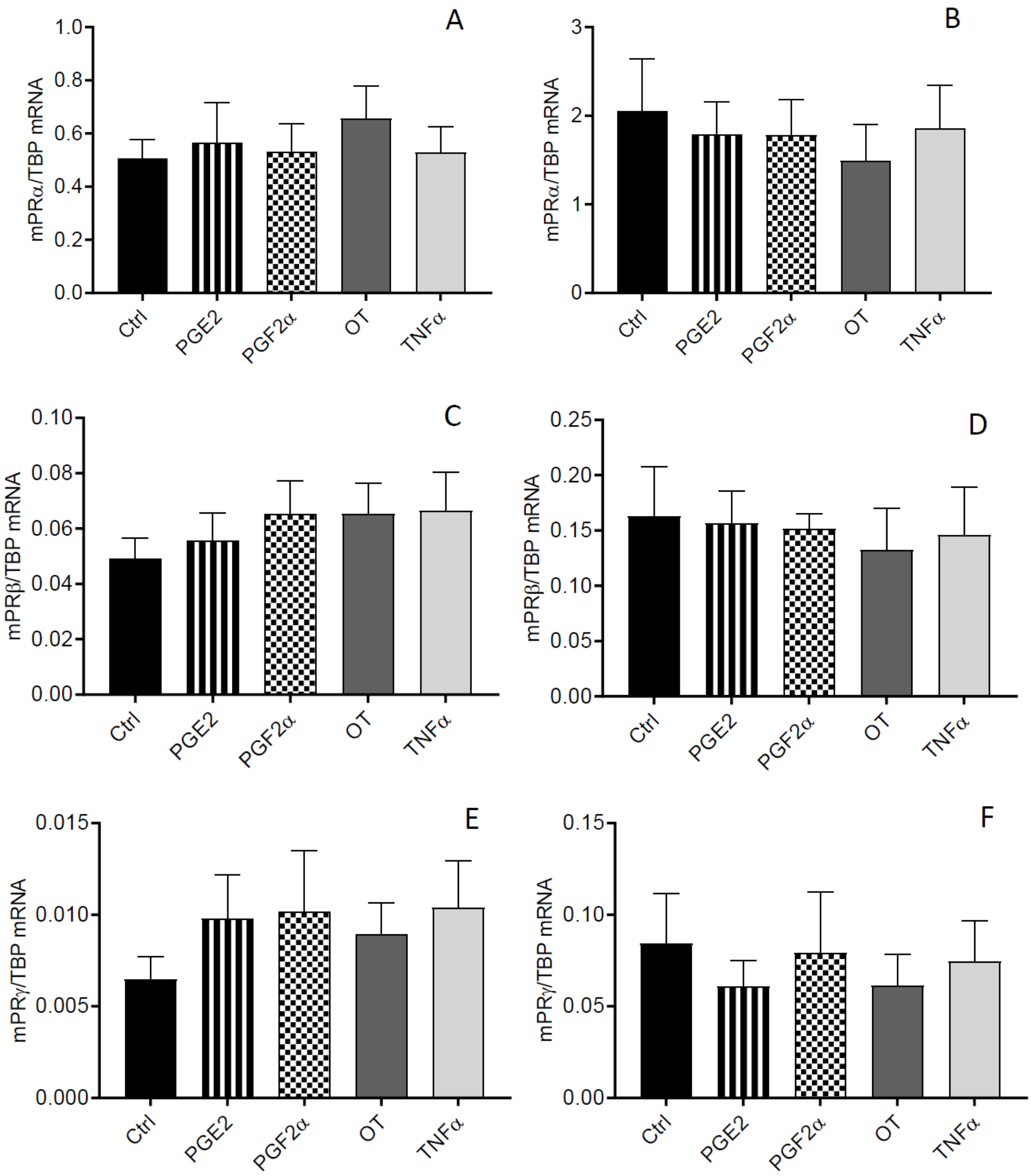

Figure S2. The influence of prostaglandin E2 (PGE2; 10-6 M), prostaglandin F2α (PGF2α; 10- 6 M), oxytocin (OT; 10-7 M), and tumor necrosis factor alpha (TNFα; 10-4 M) on the mRNA expression of membrane progestin receptor (mPR) α (A, B), mPRβ (C, D) and mPRγ (E, F) in bovine myometrial cells from days 6–10 (A, C, E) and 11–16 (B, D, F) of the estrous cycle. Gene expression was determined by quantitative real-time PCR. Results are reported as mean ± SEM (n = 5). Bars with different superscripts differ significantly ($p < 0.05$).
